# Practical Hints

**Published:** 1863-06

**Authors:** J. D. White


Practical Hints.—*By J. D. White.*—We are constantly receiving letters making inquiries in relation to special points in theory in dentistry, practice, and substances in use in our profession. Whatever opinion we may entertain at present in many things must not be considered as final in theory, or as conclusive in practice. While it is entirely out of our power to answer all such communications specially, we are trying to meet such demands as fast as we can through practical hints and otherwise. It is cheering to know that so many in our profession are anxious to be properly informed in all that relates to the advancement or improvement in our noble art. We give place to the following letter:

Calais, Me., May 2d, 1863.

Dr. J. D. White:—Permit me to make a few inquiries of you, to be answered through the *Dental Cosmos*, if you can spare time, and which might benefit others as well as myself. I would like to know what you think of "hard rubber" or "vulcanite" as a base for artificial teeth? So far as regards durability and healthfulness, is it as good as gold or platina? Since gold has commanded so much premium, I have used considerably of platinum as a substitute, considering it nearly as good, if not quite, for plates. Nothing so good as gold for plugs. Hoping you will continue your "practical hints" in the *Dental Cosmos*, and to hear something in relation to vulcanite,

I remain, respectfully yours,

J. E. Grant.

P. S.—Please say something in relation to the best material for plugging large cavities where gold cannot be used either from frailty of walls or expense. J. E. G.

Whatever may be the answers to the different questions propounded, must be taken as our own views; and where they differ with other practitioners in our art, it must not be considered as taking sides against them, as it is certainly true that some operators do and must practice differently from others, depending greatly upon the place they live in, and the kind of custom they have to operate for. We have said before, in another article some time since, that every dentist makes his own kind of practice. If he does an inferior class of work, he will have an inferior class of patients; if he does first-class work, so to speak, he will get only first-class patients. This is more true in large cities than in small towns. Hence we do not complain if we see an inferior style of work from dentists of the kind we speak of, or situated as above named; but every one ought to do first-class work, no matter where he lives, for everybody who will let him.

In a large city, a dentist can exclude patients who do not either appreciate good work, or do not wish to pay for it; but in smaller towns he must operate for nearly all classes. But it does not follow that if he is obliged to do an inferior class of work that he must call it good. A dentist in a small town ought to make as good work as if he lived in a large city, and it would keep his best patients with him. Let him impress on the minds of his patients what is proper, and what is not. It is not true that all the work that is done in large cities is good. Many patients repair to large cities and get a very inferior class of work; they could in many cases do better at home. We are asked every day, why it is that country dentists do not operate as well as city dentists? We answer, that many of them do better work than many city dentists; but from some vague reason, the city has its attraction for the patient which his own home has not, and he will be satisfied with an inferior class of work, thinking that it is the best he can do. Cities are the hotbeds of quackery in almost all things as well as of the highest grades of art, and many country people make a good many trips to the city before they find either out, or get to the right places.

With regard to the first question, "hard rubber," it is a patented article, and is used for many things besides dental purposes. We do not like it as a base for artificial teeth.* Our patients seldom use it. We make gold cases frequently to take its place, and many dentists make rubber cases to take the place of gold, and give satisfaction; but this does not determine its superiority.

An article in the May number of the *Dental Cosmos*, by Dr. Robertson, is interesting on the subject, to which we refer our correspondent, and to the published proceedings of the Pennsylvania Association of Dental Surgeons, February, 1863. The members of that society gave the subject a pretty thorough consideration, and we cannot too strongly commend the perusal of those discussions to every member of the profession. The manner in which they have been con-

*See New York Dental Journal, March, 1863.

ducted for the last few years more than sustains the highest hopes of its founders.

In answer to the second question. "Platina," except for continous gum work, is not as good as gold; it is not as durable. With relation to the question, what is "the best material for plugging large cavities, where gold cannot be used either from the frailty of the walls or expense?" we use oxychloride of zinc, and have published our views upon it on several occasions in the *Dental Cosmos.* We also use "Hill's stopping" in very young children, where the teeth will not bear the packing of gold either for want of strength of the jaw to bear the pressure, or tenderness of the teeth, and in cavities on the approximal surfaces of teeth, where it cannot be kept dry long enough for the oxy-chloride filling to harden. But it is a question whether either of those substances are often required, except to save expense; then they are allowable. We would be very happy if other contributors would answer some of these questions for us.—*Dental Cosmos.*

